# Catalpol Suppresses Proliferation and Facilitates Apoptosis of OVCAR-3 Ovarian Cancer Cells through Upregulating MicroRNA-200 and Downregulating MMP-2 Expression

**DOI:** 10.3390/ijms151119394

**Published:** 2014-10-24

**Authors:** Na Gao, Jian-Xin Tian, Yu-Hong Shang, Dan-Yi Zhao, Tao Wu

**Affiliations:** 1 Department of Obstetrics and Gynecology, First Affiliated Hospital of Dalian Medical University, Dalian 116011, China; E-Mails: nagaomr@163.com (N.G.); jianxintianmr@163.com (J.-X.T.); Yuhongshangmr@163.com (Y.-H.S.); 2 Department of Oncology, Second Affiliated Hospital of Dalian Medical University, Dalian 116027, China; E-Mail: Danyizhaomr@163.com

**Keywords:** catalpol, ovarian cancer, OVCAR-3 cell, MicroRNA-200, matrix metalloproteinase-2

## Abstract

Catalpol is expected to possess diverse pharmacological actions including anti-cancer, anti-inflammatory and hypoglycemic properties. Matrix metalloproteinase-2 (MMP-2) is closely related to the pathogenesis of ovarian cancer. In addition, microRNA-200 (miR-200) can modulate phenotype, proliferation, infiltration and transfer of various tumors. Here, OVCAR-3 cells were employed to investigate whether the effect of catalpol (25, 50 and 100 μg/mL) promoted apoptosis of ovarian cancer cells and to explore the potential mechanisms. Our results demonstrate that catalpol could remarkably reduce the proliferation and accelerate the apoptosis of OVCAR-3 cells. Interestingly, our findings show that catalpol treatment significantly decreased the MMP-2 protein level and increased the miR-200 expression level in OVCAR-3 cells. Further, microRNA-200 was shown to regulate the protein expression of MMP-2 in OVCAR-3 cells. It is concluded that catalpol suppressed cellular proliferation and accelerated apoptosis in OVCAR-3 ovarian cancer cells via promoting microRNA-200 expression levels and restraining MMP-2 signaling.

## 1. Introduction

It is known that the incidence of ovarian cancer ranks third after cervical cancer and corpus cancer, while the rate of death rate ranks first [1]. The early symptoms of ovarian cancer are hidden, and it is quite difficult to identify its phenotype. Over 70% of patients develop malignancy after diagnosis [2]. It was estimated that the survival rate of patients for five years at stage I or II of ovarian cancer is 70%–90%, of which many patients could be cured only by surgery and the survival rate of patients for five years at stage III or IV of ovarian cancer is only 20% [3].

In recent years, numerous studies clarified that extracellular matrix (ECM) degradation, a mechanism of tumor cells, suggests that ovarian cancer infiltrates normal tissues and triggers the transfer [4]. Matrix metalloproteinase (MMP) is the main factor for tumor infiltration and transfer, as it is the major enzyme for ECM degradation, which can degrade all collagen-forming matrix and basement membrane, protein proteoglycan and laminin and other elements [5]. Besides, MMP-2 may be also involved in the occurrence, infiltration and transfer of ovarian cancer, and one of the molecular markers for ovarian tumor progression and molecular targets in ovarian cancer [6].

MicroRNAs (miR) are an abundant type of small (70 to 100 bases) non-coding RNAs with regulatory functions, and play a key role in many human disorders, including cancer. It has previously been shown that miR regulates nearly 30 percent of human genes [7]. They are closely associated with cell synthesis period regulation, blood cell production, fat metabolism, organogenesis, cell proliferation, cell differentiation and apoptosis [8]. Indeed, miR-200 was reported to regulate cellular proliferation, infiltration and transfer of various tumor cells [9]. Kobayashi *et al.* demonstrated that miR-200 family transcripts could be identified in human ovarian cancer OVCAR-3 cells [10].

Catalpol is an iridoid glucoside compound and has biological activities including anti-cancer, neuroprotective and anti-inflammatory properties [11]. *In vitro*, catalpol can protect lipopolysaccharide-induced acute lung injury via inhibition of TNF-α, IL-6, IL-4 and IL-1β, and up-regulated IL-10 [12]. In addition, catalpol protected against cerebral ischaemia/reperfusion injury in gerbils through reducing free radicals and suppressing lipid peroxidation [13]. Meanwhile, a previous report was shown to protect dopaminergic neurons against inflammation-related neurodegenerative diseases after catalpol treatment [14]. In a recent *in vitro* study, catalpol was a Taq DNA polymerase inhibitor and showed a marginal growth inhibition against the human ovarian cancer A2780 cell [15]. However, whether catalpol influences human ovarian cancer remains to be elucidated. Besides, there is no relevant publication on the potential antitumor mechanism of catalpol in ovarian cancer. Our current investigation was conducted to evaluate the curative effect of catalpol against ovarian cancer cells and explore whether its anticancer property is associated with modulations of miR-200 and MMP-2.

## 2. Results

### 2.1. MTT Analysis and Caspase-3 Activity Measurement

**Figure 1:**
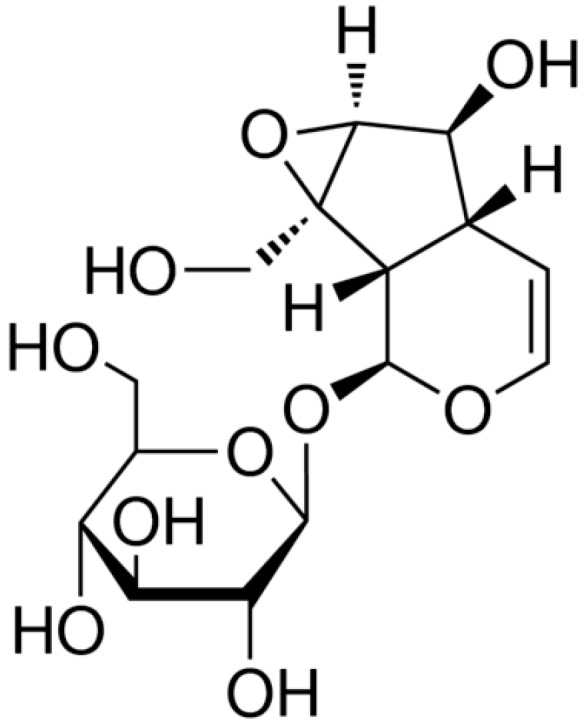
The chemical structure of catalpol.

**Figure 2:**
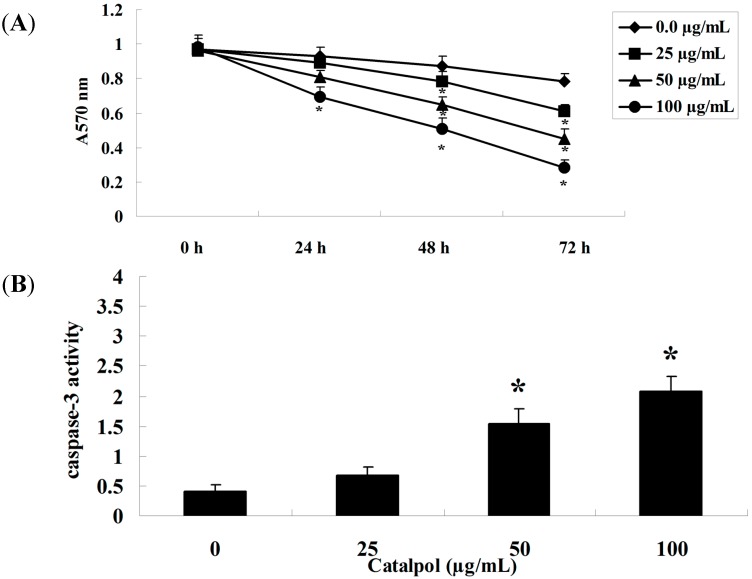
The effect of catalpol on OVCAR-3 cell growth. Catalpol treatment dose-dependently inhibited the viability of OVCAR-3 cells (**A**) and activity of caspase-3 was significantly enhanced after treatment (**B**). * *p* < 0.01 compared with 0 mg/mL catalpol treatment group.

The chemical structure of catalpol is shown in Figure 1. Notably, the effects of catalpol on the viability of OVCAR-3 cells were time and concentration-dependent. We tested a range of concentrations (25–100 μg/mL) [16] at three different time points. Catalpol (50 and 100 μg/mL) significantly inhibited the proliferation of OVCAR-3 cells at 48 and 72 h, with statistical significance (Figure 2A). Meanwhile, treatment with catalpol (0, 25, 50 and 100 μg/mL) could significantly increase caspase-3 activity in OVCAR-3 cells (Figure 2B).

### 2.2. Flow-Cytometric Analysis for Detecting Cellular Apoptosis

**Figure 3:**
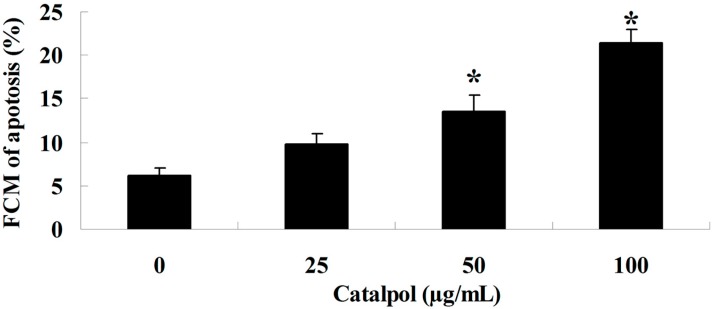
The apoptosis by catalpol. * *p* < 0.01 compared with 0 mg/mL catalpol treatment group.

The effect of catalpol was assessed in OVCAR-3 cells given catalpol (0, 25, 50 and 100 μg/mL). As shown in Figure 3A,B, adding catalpol (0, 25, 50 and 100 μg/mL) for 48 h induced a concentration-dependent apoptosis of OVCAR-3 cells. However when catalpol (50 and 100 μg/mL) was used for 48 h, apoptosis of OVCAR-3 cells was significantly decreased with statistical significance.

### 2.3. Inhibition of Catalpol on MMP-2

**Figure 4:**
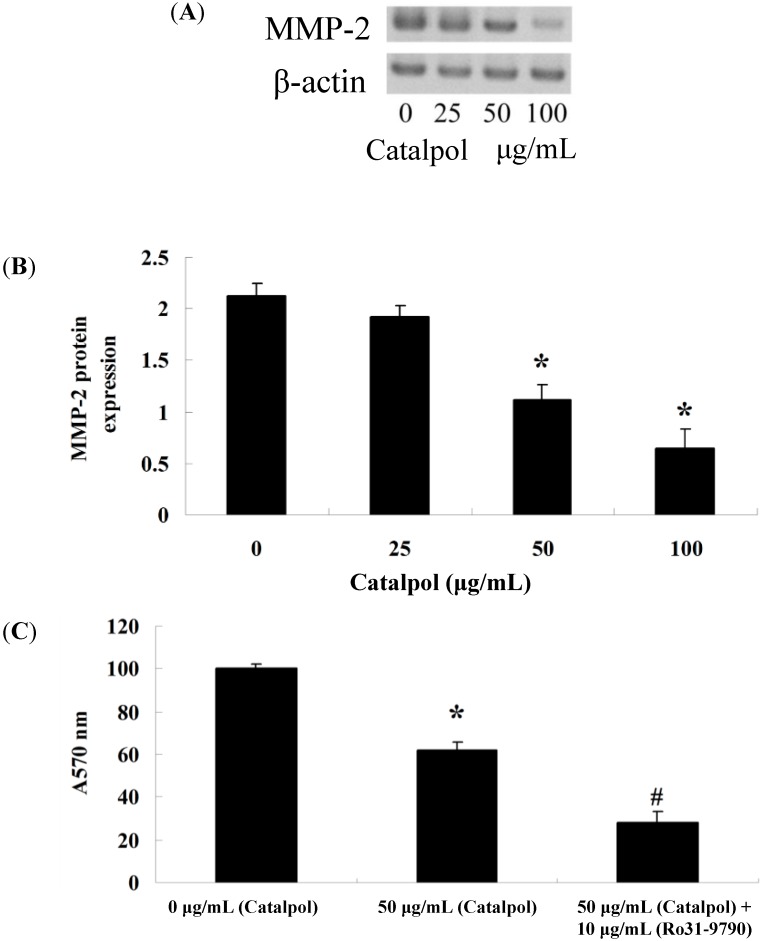
The effects of catalpol on MMP-2. MMP-2 activity was dose-dependently reduced in OVCAR-3 cells after treatment with catalpol for 48 h by gelatin zymography assays (**A**); statistical analysis of MMP-2 protein level was calculated (**B**) and the cell viability (**C**). * *p* < 0.01 compared with 0 mg/mL catalpol treatment group, # *p* < 0.01 compared with 50 mg/mL catalpol treatment group.

After treating with catalpol (0, 25, 50 and 100 μg/mL) for 48 h, we used gelatin zymography assays to analyze the MMP-2 protein level in OVCAR-3 cells (Figure 4A). Certainly, when catalpol (50 and 100 μg/mL) was used for 48 h, the MMP-2 protein levels in OVCAR-3 cells were significantly decreased with statistical significance (Figure 4B). An MMP-2 inhibitor was used to analyze the effect on cell viability; after treatment with catalpol (50 μg/mL) and MMP-2 inhibitor (Ro31-970) at 48 h, cell viability was detected using MTT. Interestingly, when catalpol (50 μg/mL) was used for 48 h, cell viability was significantly decreased, compared to that of the control-group (Figure 4C). However, when catalpol (50 μg/mL) and Ro31-970 (10 μg/mL, Roche, Mannheim, Germany) were used for 48 h, cell viability was also dramatically reduced, compared to that of the catalpol-group (Figure 4C).

### 2.4. Catalpol Activates miR-200 Expression

**Figure 5:**
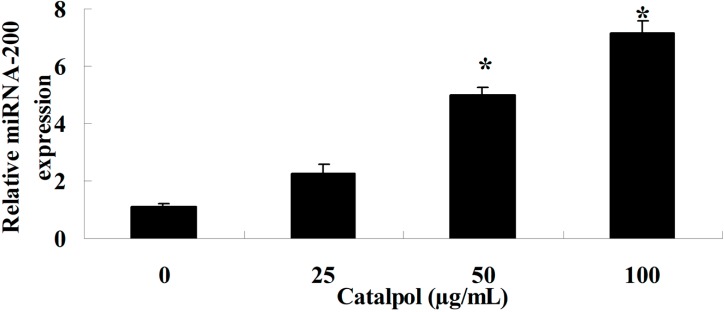
Catalpol induced miR-200 expression. * *p* < 0.01 compared with 0 mg/mL catalpol treatment group.

The intracellular miR-200 expression level was determined with Q-PCR analysis, with treatment with catalpol (0, 25, 50 and 100 μg/mL) for 48 h. As shown in Figure 5, the miR-200 expression level was significantly increased with statistical significance (Figure 5).

### 2.5. Overexpression of miR-200 and MMP-2 Expression

**Figure 6:**
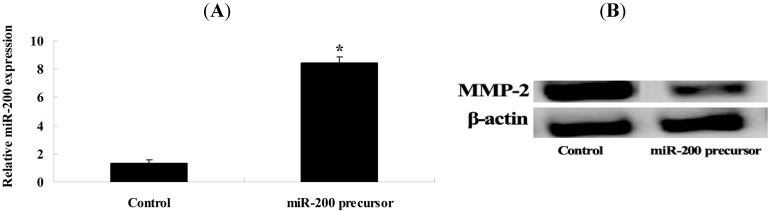
Overexpression of miR-200 can suppress MMP-2 expression. MiR-200 precursor significantly accelerated the expression of miR-200 (**A**); Transfection of miR-200 precursor inhibited MMP-2 protein expression (**B**). * *p* < 0.01 compared with control group.

We transfected miR-200 precursor into OVCAR-3 cells to further investigate whether miR-200 regulates MMP-2 expression in OVCAR-3 cells. The MMP-2 protein level of OVCAR-3 cells was analyzed. Our results indicate that miR-200 precursor could significantly elevate the expression of miR-200 (Figure 6A) and obviously inhibit MMP-2 expression in OVCAR-3 cells (Figure 6B).

### 2.6. Anti-miR-200 Can Reverse the Effect of Catalpol

**Figure 7:**
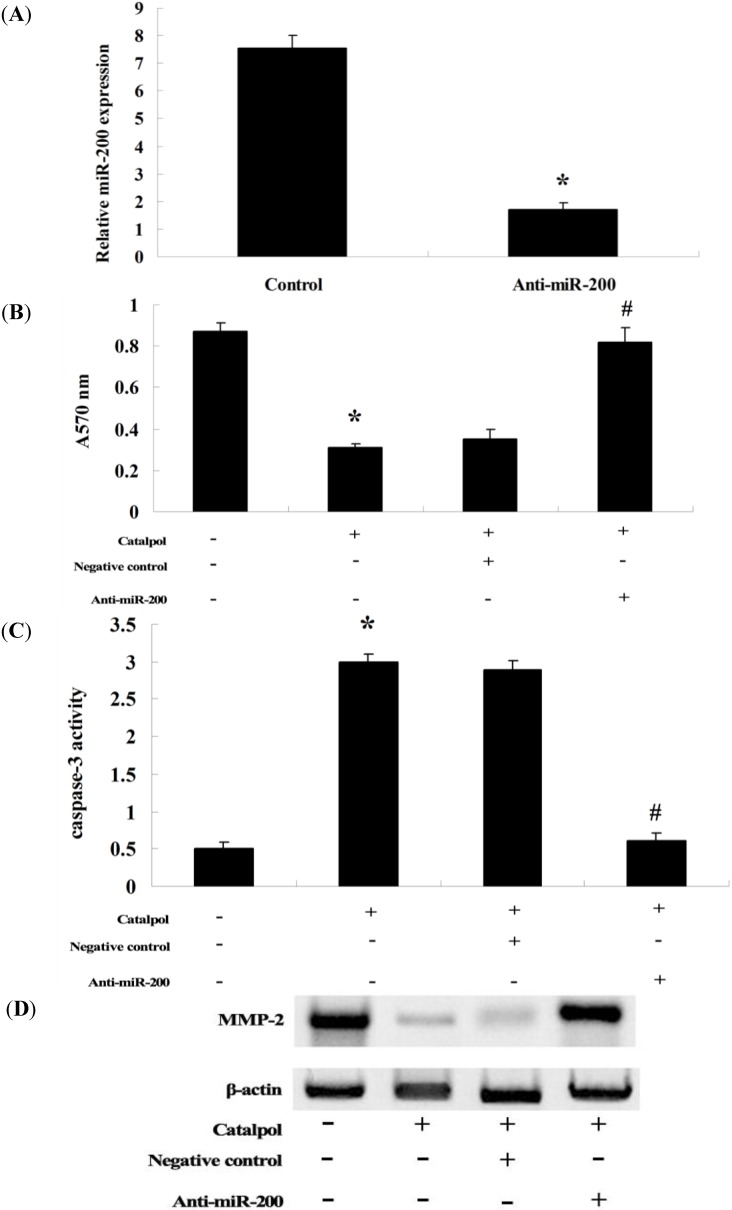
Anti-miR-200 can reverse the effect of catalpol. Anti-miR-200 could evidently decrease the expression of miR-200 in OVCAR-3 cells (**A**); After treatment with catalpol (50 μg/mL) for 48 h, anti-miR-200 significantly promoted the cell proliferation of OVCAR-3 cells (**B**); After treatment with catalpol (50 μg/mL) for 48 h, anti-miR-200 evidently inhibited cell apoptosis of OVCAR-3 cells (**C**) and the anti-miR-200 significantly increased MMP-2 activity in OVCAR-3 cells after catalpol (50 μg/mL) treatment at 48 h (**D**). * *p* < 0.01 compared with 0 mg/mL catalpol treatment group, and # *p* < 0.01 compared with catalpol-treated group transfected with negative control.

The effect of catalpol was assessed in OVCAR-3 cells given the standard pretreatment with catalpol (50 μg/mL, 48 h). In the same series of experiments, we used anti-miR-200 antibody to evaluate the role of miR-200 in catalpol inhibition of OVCAR-3 cells. Our results indicate that transfection of anti-miR-200 antibody penetrated into OVCAR-3 cells and significantly reduced the expression of miR-200 in OVCAR-3 cells (Figure 7A). Anti-miR-200 antibody could significantly reduce catalpol’s effect on cell proliferation (Figure 7B), and its apoptotic effect in OVCAR-3 cells (Figure 7C), neutralized the inhibitory effect through downregulating MMP-2 activity in OVCAR-3 cells.

## 3. Discussion

The incidence of ovarian cancer ranks sixth in the world for female cancer, and due to a lack of effective methods for early diagnosis, more than 70% of patients are in advanced stages with poor prognosis after diagnosis [17]. According to the data of GLOBOCAN 2008 released by the International Agency for Research on Cancer (IARC), the world average incidence rate of ovarian cancer of 184 countries in the world in 2008 is 6.3/100,000, and China is 6.0/100,000, which ranks No. 85 in the world and is at the medium level [18]. In the present study, catalpol could markedly reduce the proliferation of OVCAR-3 cells. These results showed that catalpol has antitumor activity for ovarian cancer cell lines. Studies have reported the naturally occurring iridoid catalpol is a Taq DNA polymerase inhibitor and inhibited ovarian cancer *in vitro* [15].

Catalpol has wide pharmacological effects and is expected to serve as a new type of therapeutic drug. DNA polymerase is a target with an important role for anticancer treatments. Catalpol has an obvious effect on inhibiting heat-resistant DNA polymerase. In the measurement of hyperchromic effect of double-stranded DNA, catalpol did not affect the thermal denaturation curve, and the inhibitory effect of catalpol was dependent on the concentration of DNA; the increase in dNTP concentration can restore the activity of heat-resistant DNA polymerase, and the results suggested that catalpol combined with the target of heat-resistant DNA polymerase through dNTPs competition, thus inhibiting this enzyme and exerting an anticancer role [15,19,20]. Results from our current study indicate that catalpol treatment could effectively accelerate the apoptosis of OVCAR-3 cells and increase the activity of caspase-3 in OVCAR-3 cells.

MMP2 is not only of great significance in tumor infiltration and transfer, but also plays a role in tumor angiogenesis. MMP2 has a dual role in tumor infiltration, transfer and angiogenesis, which helps to determine patients’ sensitivity during traditional treatment, and has inspired efforts to develop bio-specific therapy for tumors [21]. In the present study, it was demonstrated that catalpol stimulation of OVCAR-3 cells significantly restrained the expression of MMP-2 protein. Recent studies showed that the 37-kDa laminin receptor precursor mediates GnRH-II-induced MMP-2 expression in OVCAR-3 cells [22].

The functional role of miRNAs in proliferation, infiltration and transfer for epithelial tumors has become a research hotspot in recent years. A number of studies have found a highly significant increase in expression level of the miR-200 family in ovarian cancer tissue [23]. Our study found that catalpol could activate miR-200 expression level in OVCAR-3 cells. Of interest, we found that overexpression of miR-200 could significantly promote the expression of miR-200 and obviously restrain MMP-2 expression in OVCAR-3 cells. Meanwhile, anti-miR-200 could promote the expression of MMP-2 in OVCAR-3 cells. Lastly, anti-miR-200 antibody could significantly reduce the effect of catalpol on cell proliferation and apoptosis of OVCAR-3 cells. In contrast, a previous report indicated that miR-200a, miR-200b and miR-200c overexpression could promote the aggressive tumor progression in patients with epithelial ovarian cancer. The appearance of this discrepancy is due to the disease stages and the differences of clinical and *in vitro* investigations. Collectively, this result illustrated that miR-200 can regulate and control MMP-2 expression in OVCAR-3 cells. A previous work revealed that overexpression of the miR-200 family can increase the level of MT1-MMP in pancreatic cancer [24]. These studies suggest that overexpression of miR-200a, miR-200b and miR-200c may promote the aggressive tumor progression in patients with epithelial ovarian cancer [23].

In conclusion, the present study revealed that catalpol has antitumor activity for ovarian cancer cell lines and this effect may be associated with promoting the expression of miR-200 and restraining the expression of the MMP-2 protein signaling pathway.

## 4. Experimental Section

### 4.1. Chemicals and Reagents

Catalpol (with a purity ≥96%) was purchased from Sigma Aldrich (Sigma, Sigma Aldrich, Carlsbad, CA, USA). Roswell Park Memorial Institute-1640 (RPMI-1640) and fetal calf serum (FBS) were obtained from Gibco Hyclone (Invitrogen Company, Carlsbad, Australia) and (Invitrogen Company, BRL, Carlsbad, CA, USA), respectively. 3.3-(4,5-dimethylthiazol-2-yl)-2,5-diphenyltetrazolium bromide (MTT) was purchased from Sigma Chemical Corporation (Sigma, Sigma Aldrich, CA, USA). Annexin V-FITC/PI Apoptosis Detection kit was obtained from BeastBio (Shanghai, China). Ro31-9790 was purchased from Roche (Boehringer, Germany). cDNA Synthesis kit and the SYBR Green kit were obtained from Tiangen (Beijing, China).

### 4.2. Cell Culture

The human ovarian cancer, OVCAR-3 cell line was cultured in RPMI-1640 containing 10% (*v*/*v*) FBS with 100 U penicillin/streptomycin. The cells were incubated in a humidified atmosphere containing 5% CO_2_ at a temperature of 37 °C. OVCAR-3 cells were treated with catalpol in complete RPMI-1640 medium.

### 4.3. Cell Viability Assay

OVCAR-3 cells were cocultured with catalpol (25, 50 and 100 μg/mL) for 48 h. OVCAR-3 cell viability was determined using the MTT assay. For MTT assays, each well was given 10 µL MTT (5 mg/mL, Sigma) and incubated for 4 h at 37 °C in a humidified atmosphere of 5% CO_2_. The culture medium was removed and 150 µL dimethyl sulfoxide (DMSO) was added to each well for 10 min at room temperature whilst being shaken. The absorbance was determined with an ELISA reader at 490 nm.

### 4.4. Caspase-3 Activity Assays

OVCAR-3 cells were treated with catalpol (25, 50 and 100 μg/mL) for 48 h. The activity of caspase-3 was detected by the caspase-3 colorimetric assay kit (Sangon Biotech, Shanghai, China) according to the manufacturer’s protocol. An equal amount of total protein extract was incubated at 37 °C with either Ac-IETD-pNA or Ac-LEHD-pNA for caspase-3 assay for 4–6 h. The fluorescence was detected at the wavelength of 405 nm.

### 4.5. Flow Cytometry

For detection of apoptosis, annexin V-FITC/PI Apoptosis Detection kit (BeastBio, Shanghai, China) was used. OVCAR-3 cells were cocultured with catalpol (25, 50 and 100 μg/mL) for 48 h. Following the manufacturer’s protocol, apoptosis of OVCAR-3 cells were detected using an annexin V-FITC/PI Apoptosis Detection kit and observed through flow cytometry.

### 4.6. Gelatin Zymography Assays of MMP-2

Gelatin zymography was performed as described for the MMP-2 protein level of OVCAR-3 cells. Briefly, 20–30 µL media were collected and were added into new centrifuge tubes. Then an equal volume of sodium dodecyl sulfate (SDS) sample buffer was added. The miscible liquids were subjected to 10% SDS-PAGE electrophoresis gel impregnated with 0.1% gelatin. After electrophoresis, the gel was washed three times for 20 min totally at room temperature in 2.5% Triton X-100 to remove SDS, and incubated in a reaction buffer at 37 °C for 12 h. After incubation, the gel was stained with 0.05% Coomassie brilliant blue R-250 (Amresco, Solon, OH, USA).

### 4.7. Q-PCR Analysis of miR-200 Expression

OVCAR-3 cells were plated in 6 well/plates and incubated for 24 h. Then cells (2.0 × 10^5^ cells/mL) were treated with different concentrations of catalpol (0, 0.5, 1 and 2 mg/mL). According to the manufacturer’s protocol, cDNAs were synthesized using cDNA Synthesis kit (Tiangen, Beijing, China) and the miR-200 expression level were detected through SYBR Green kit (Tiangen, Beijing, China). Cycling conditions were as follows: 95 °C for 10 min followed by 32 cycles at 94 °C for 10 s, 58 °C for 25 s and 72 °C for 30 s, and then 72 °C for 10 min. For miR-200, the primer was as follows: 5'-TGCATCATTACCAGGCAGTATTAGA-3', 5'-CCTCTTACCTCAGTTACAATTTATA-3', respectively.

### 4.8. Transfection of miR-200 and Anti-miR-200

The miR-200 precursor and anti-miR-200 (Ambion, Carlsbad, CA, USA) were synthesized and purchased from Sangon Biotech (Shanghai, China). Briefly, 100 nmol/L of miR-200 or anti-miR-200 was transfected into OVCAR-3 cells with lipofectamine 2000 (Invitrogen, Carlsbad, CA, USA). After transfection for 24 h, cells were treated with different concentration of catalpol (0, 25, 50 and 100 μg/mL).

### 4.9. Statistical Analysis

All studies conducted were performed with SPSS 17.0 software. Differences were analyzed using Student’s *t*-test. Data are presented as means ± S.D. Values of *p* < 0.05 were considered statistically significant.

## 5. Conclusions

In conclusion, we have provided evidence that catalpol suppresses proliferation and facilitates apoptosis of OVCAR-3 ovarian cancer cells through upregulation of microRNA-200 and downregulation of MMP-2 expression.
